# Long non-coding RNA profile in mantle cell lymphoma identifies a functional lncRNA ROR1-AS1 associated with EZH2/PRC2 complex

**DOI:** 10.18632/oncotarget.17956

**Published:** 2017-05-17

**Authors:** Guangzhen Hu, Shiv K. Gupta, Tammy P. Troska, Asha Nair, Mamta Gupta

**Affiliations:** ^1^ Division of Hematology, Mayo Clinic Rochester, Rochester, MN, USA; ^2^ Department of Radiation Oncology, Mayo Clinic Rochester, Rochester, MN, USA; ^3^ Division of Biomedical Statistics and Informatics, Mayo Clinic Rochester, Rochester, MN, USA; ^4^ Department of Biochemistry and Molecular Medicine, School of Medicine and Health Sciences, The George Washington University, GW Cancer Center, Washington, DC, USA

**Keywords:** lncRNA ROR1-AS1, MCL, PRC2, EZH2

## Abstract

Mantle cell lymphoma (MCL) is an aggressive B-cell lymphoma characterized by rapid disease progression. The needs for new therapeutic strategies for MCL patients call for further understanding on the molecular mechanisms of pathogenesis of MCL. Recently, long noncoding RNAs (lncRNAs) have been recognized as key regulators of gene expression and disease development, however, the role of lncRNAs in non-Hodgkin lymphoma and specifically in MCL is still unknown. Next generation RNA-sequencing was carried out on MCL patient samples along with normal controls and data was analyzed. As a result, several novel lncRNAs were found significantly overexpressed in the MCL samples with lncRNA ROR1-AS1 the most significant one. We cloned the ROR1-AS1 lncRNA in expression vector and ectopically transfected in MCL cell lines. Results showed that overexpression of ROR1-AS1 lncRNA promoted growth of MCL cells while decreased sensitivity to the treatment with drugs ibrutinib and dexamethasone. ROR-AS1 overexpression also decreased the mRNA expression of P16 (*P* = 0.21), and SOX11 (*p* = 0.017), without much effect on P53, ATM and P14 mRNA. RNA-immunoprecipitation assays demonstrated high affinity binding of lncRNA ROR1-AS1 with EZH2 and SUZ12 proteins of the polycomb repressive complex-2 (PRC2). Suppressing EZH2 activity with pharmacological inhibitor GSK343 abolished binding of ROR1-AS1 with EZH2. Taken together, this study identified a functional lncRNA ROR-AS1 involved with regulation of gene transcription via associating with PRC2 complex, and may serve as a novel biomarker in MCL patients.

## INTRODUCTION

Mantle cell lymphoma (MCL) is an aggressive B-cell malignancy usually diagnosed as a late-stage disease with patients above 60 years old. Although the median survival of MCL patients has increased with the development of new therapeutic strategies [1, 2], MCL has been regarded as an incurable blood cancer that is characterized by rapid disease progression. The constitutive overexpression of cyclin D1 caused by t(11;14) (q13;q32) translocation is considered to be the initial oncogenic steps for the development of MCL [3]. Certain key genes in cell cycle pathway (INK4a, RB1, and ARF), and in DNA damage response pathways (ataxia telangiectasia mutated (ATM and TP53) are frequently deregulated, deleted or mutated [4–7]. More recently, the transcription factor SOX11 has been identified as a specific marker for both Cyclin D1 positive and negative MCL and regarded as a key gene in the pathogenesis of MCL [8, 9].

Although the clinical outcome of MCL patients has been improved with the adjustment of the treatment protocol according to the heterogeneous biology and clinical presentation, relapses and progressive resistance to treatment are common. Therefore, new therapeutic strategies based on understanding of the molecular mechanism of pathogenesis are in great need. Long noncoding RNAs (lncRNAs) are RNA transcripts with a length ranging from 200 bases to more than 20 kilo base that lack protein-coding capacity, and may have different origins related to coding gene in chromosome such as intronic, exonic, intergenic, intragenic, promoter regions, enhancer sequences and antisense strand [10]. Unlike protein coding RNA transcripts, which are exported to the cytoplasm, most of the lncRNAs are retained within the nucleus. LncRNAs retained in the nucleus are potentially targeted for nuclear genome surveillance and transcription machinery. Some of the key biological processes regulated through lncRNAs include epigenetic and transcriptional regulation of gene expression [11, 12], post-transcriptional regulation such as mRNA processing, and translation [13–18], cell differentiation and development [19] and human disease especially the cancer [20]. LncRNA profile in B cell lymphoma and specifically in MCL has not been identified or mechanistically studied. In this study, we aimed to identify the lncRNAs deregulated in MCL and explore their role in MCL pathogenesis.

## RESULTS

### Identification of lncRNAs profile in MCL by next generation RNA-sequencing

**Figure 1:**
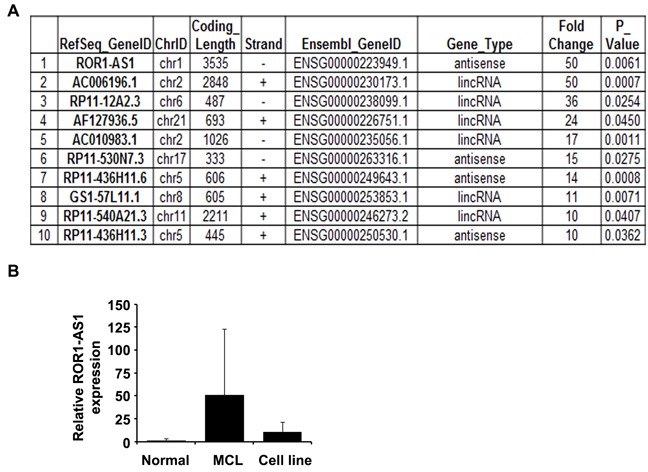
LncRNA profile in MCL **A.-B.** LncRNAs profile in MCL patient samples identified by next generation RNA-sequencing. Top 10 LncRNAs expression and their characteristics in cells from MCL patient versus non-lymphoma control (*n* = 5) are shown. (B) Confirmation of ROR1-AS1 lncRNAs in MCL samples and cell lines was done by QRT-PCR using specific primers. Bar diagram showing mean of ROR1-AS1 expression in normal controls (*n* = 5), MCL patient samples (*n* = 5) and MCL cell lines (*n* = 4).

To identify lncRNAs profile in MCL, we performed next generation RNA-sequencing and compared the lncRNA expression in MCL patient samples with non-lymphoma control samples. For sorted lncRNAs, we used log2 fold changes greater than 10 and p-value less than 0.05. Based on these criteria we identified ten different lncRNAs upregulated in MCL patient samples with ROR1-AS1 (also called RP11-24J) on the top of the list (Figure 1A). To validate findings from RNA-sequencing, expression of ROR1-AS1 in patient samples and normal controls was further analyzed by quantitative RT-PCR. Results show that ROR1-AS1 is overexpressed (more than 50 folds) in most of the MCL tumor samples as compared to the normal controls (Figure 1B), although due to the small sample size p value was not significant (*p* = 0.16). Furthermore, MCL cell lines (Mino, Granta, JVM2 and Z138) also express higher levels of ROR1-AS1 as compared to normal controls, although the expression in MCL cell lines was relatively low as compared to the MCL patient samples. Collectively, we have identified lncRNA transcripts upregulated in MCL patient samples with ROR1-AS1 as prominent candidate.

### LncRNAs ROR1-AS1 and AC006196 are predominantly localized in the nucleus

**Figure 2:**
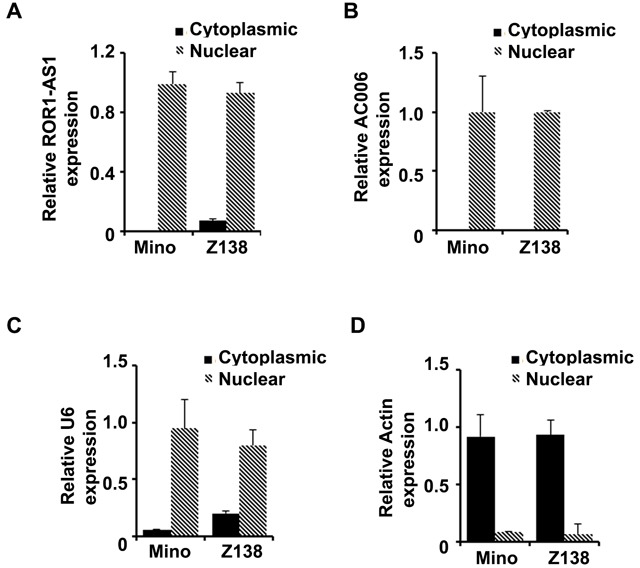
Subcellular distribution of lncRNAs ROR1-AS1 and AC006196 in MCL **A.-D.** Bar graphs represent relative RNA quantity in cytosolic versus nuclear fraction for the lncRNA ROR1-AS1 (A), AC006196.1 (B) in Mino and Z138 cell lines, levels of U6 mRNA (C) and Actin mRNA (D) were used as controls to check purity of the nuclear and cytosolic fractions, respectively. Cytoplasmic RNA and nuclear fraction of RNA were extracted, and the expression of each the RNA quantified by QPCR. Experiment was repeated 2 times with similar results.

One of the important factors in understanding the function of lncRNAs is their intracellular location. We separated cytosol RNA from nuclear RNA and checked the localization of the lncRNAs ROR1-AS1 and AC006196 in MCL cell lines, Mino and Z138. QRT-PCR data from fractionated RNA species clearly showed that lncRNA ROR1-AS1 and AC006196 found in Mino and Z138 cells were predominantly localized within the nucleus (Figure 2A-2B). To ensure purity of fractionation β-actin and U6 mRNA were assessed as markers of cytosolic and nuclear RNA transcripts, respectively. As shown in Figure 2C-2D, about 90% of β-actin transcripts in both Mino and Z138 cell lines were in the cytosolic fraction while 80-90% of U6 mRNA transcripts localized in the nuclear fraction (Figure 2C-2D). These results indicate that lncRNA ROR1-AS1 and AC006196 are localized in the nucleus and likely to be involved in epigenetic regulation of gene transcription rather than protein translation or transport.

### The biological functions of the MCL associated lncRNAs

**Figure 3:**
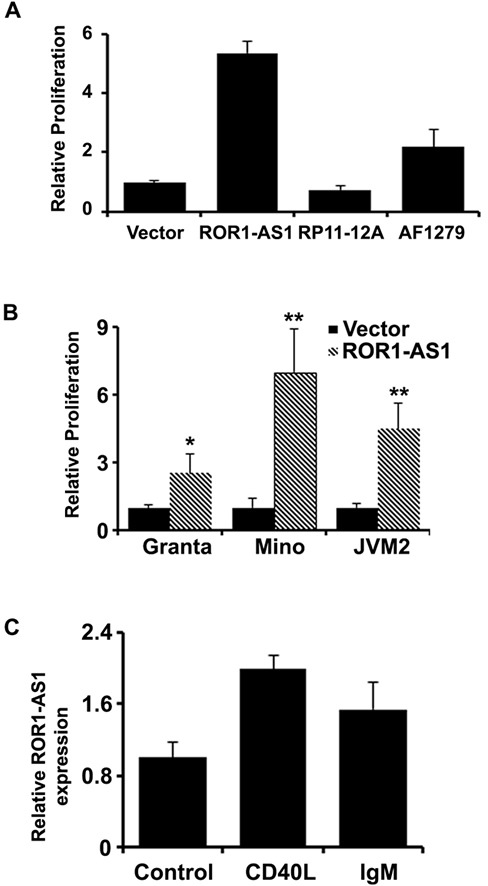
The effect of lncRNAs overexpression on cell growth **A.-B.** Bar graphs showing relative ^3^H-thymidine incorporation as a measure of proliferation in Mino cells transfected with empty vector, or vector with expression cassette for lncRNAs, ROR1-AS1, RP11-12A2.3 or AF127936.5. Bars represent mean of 4 independent measurements and compared by t-test, ROR1-AS1 (*p* = 0.0000005), RP11-12A2.3 (*p* = 0.01), AF127936.5 (*p* = 0.006). (B) Bar graphs depicting effects of the ROR1-AS1 on proliferation (^3^H-thymidine incorporation) in Mino, Granta, and JVM2 cells. Bars represent mean of 3 independent measurements and compared by t-test, Mino (*p* = 0.000007), Granta (*p* = 0.00007), JVM2 (*p* = 0.00006). **C.** The effect of CD40L (*p* = 0.002) and IgM (*p* = 0.02) on the expression of ROR1-AS1 in CD19+ normal B cells by QPCR.

We next tested if the MCL associated lncRNAs (as shown in Figure 1A) have any biological relevance or affect the growth of the MCL cells. We cloned 3 lncRNAs ROR1-AS1, RP11-12A and AF12791 into mammalian expression vector, pcDNA3.1. Transient transfection with lncRNA expression vectors in MCL cell line Mino increased expression of corresponding lncRNAs several hundred folds over the base line (Supplementary Figure 1A-1B). As shown in Figure 3A, overexpression of RP11-12A in Mino cells had no significant impact on cell proliferation, while overexpression of lncRNAs ROR1-AS1 and AF127936.5 enhanced ^3^H-thymidine uptake by 5 and 2 folds, respectively (Figure 3A). Thus, our results suggest that lncRNA ROR1-AS1 may have an oncogenic effect in MCL cells. To further validate this notion, the effect of ROR1-AS1 overexpression was tested in two more MCL cells lines, Granta and JVM2. Consistently overexpression of ROR1-AS1 in both of additional MCL cell lines was associated with increased ^3^H-thymidine uptake (Figure 3B). On contrary, silencing the expression of ROR1-AS1 by siRNA decreased ^3^H-thymidine in Granta cell line as compared to non-targeting siRNA controls (Supplementary Figure 2A-2B). Taken together, these results suggest that deregulation of lncRNA in MCL patient samples is in part responsible for sustained growth of MCL cells.

B cell receptor (BCR) signaling is very important in B-cell development and maturation and the pathogenesis of B cell lymphoma including MCL [21]. To explore whether the lncRNAs are involved in BCR signaling, we isolated B cells from peripheral blood with CD19 antibody and treated with CD40L and IgM. QRT-PCR data showed that lncRNA ROR-AS1 was induced with the treatment of CD40L and IgM (Figure 3C). These data indicated the lncRNA ROR-AS1 is likely involved in the B cell receptor signaling.

### The interaction of lncRNA ROR-AS1 with polycomb repressive complex

**Figure 4:**
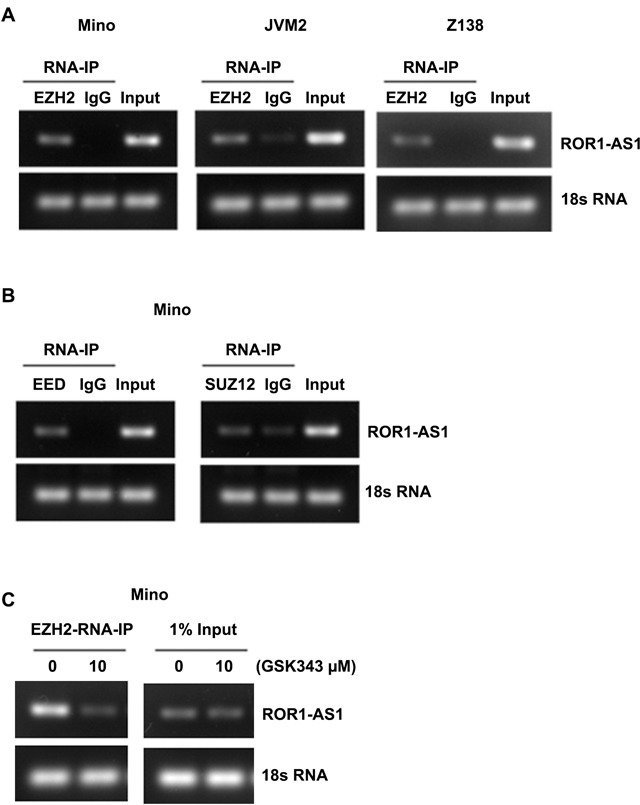
Analysis of interaction between lncRNA ROR1-AS1 and PRC2 complex **A.-C.** Representative gels pictures showing enrichments of ROR1-AS1 after RNA-IP with EZH2 antibody or IgG in lysates of Mino, Granta and Z138 cells, 1% of input was analyzed as qualitative control and 18S RNA was used as loading control. (B) Gels pictures showing enrichment of ROR1-AS1 after RNA-IP with EED or SUZ12 antibodies or IgG in lysates of Mino cells, 1% of input and 18S RNA are shown as controls. C) Gel pictures showing effect of EZH2 inhibition on enrichments of ROR-AS1 after RNA-IP with EZH2 antibody. RNA-IPs were performed on lysates from Mino cells pretreated with and without 10 μM GSK343, 1% of input was analyzed as qualitative control and 18S RNA was used as loading control.

LncRNAs especially those localized with in the nucleus have been previously linked to the epigenetic control of transcriptional regulation through their association with chromatin remodeling factors such as polycomb repressive complex 2 (PRC2) [22]. We examined whether ROR1-AS1 physically associates with the PRC2 subunits EZH2, SUZ12 and EED. Results from RNA-immunoprecipitation (RNA-IP) with antibody to EZH2 and subsequent analysis demonstrate a high affinity binding of lncRNA ROR1-AS1 with EZH2 in three different MCL cell lines (Figure 4A). Furthermore, RNA-IP performed with antibodies to EED and SUZ12 also showed binding of ROR1-AS1 lncRNA with EED and SUZ12 (Figure 4B). These results suggest that ROR1-AS1 interacts with PRC2, whether interaction between ROR1-AS1 and PRC2 depends on PRC2 activation was however, was unclear. To address this question, we used GSK343, a potent and selective inhibitor of the EZH2 activity [23]. As shown in Figure 4C, interaction between ROR1-AS1 and EZH2 was blocked in cells pretreated with GSK343, while a robust binding occurred in untreated controls (Figure 4C). Collectively, these data suggest that lncRNA ROR1-AS1 interaction between lncRNA ROR1-AS1 and PRC2 complex may be involved in the epigenetic regulation of gene transcription in MCL cells.

### Effect of ROR1-AS1 on the key genes involved in MCL pathogenesis

**Figure 5:**
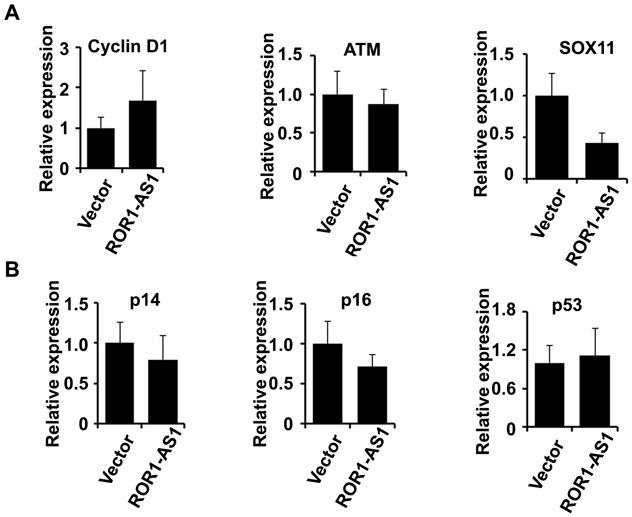
The effect of lncRNA ROR1-AS1 overexpression on transcription of key genes in MCL **A.-B.** Bar graphs depicting relative mRNA transcript of cyclin D1, ATM, SOX11 (A) and (B) p14, p16, p53 in Mino cells transfected with empty vector, or vector with expression cassette for lncRNAs ROR1-AS1. mRNA transcripts were analyzed by Q-RTPCR using gene specific primers. Each bar represent mean of 4 independent measurements and comparisons are based on paired t-test, (A) cyclin D1 (*P* = 0.21), ATM (*P* = 0.67), SOX11 (0.017); (B) p14 (*P* = 0.41), p16 (*P* = 0.21), p53 (*P* = 0.73).

To study the functional role of lncRNA ROR1-AS1 in MCL cells, we checked if overexpression of ROR1-AS1 modulates the expression of key genes involved in the pathogenesis of MCL [3, 24]. We specifically assessed the effect of ROR1-AS1 overexpression on cyclin D1, p14 (ARF), p16 (INK4a), ATM, p53 and SOX11. QRT-PCR data showed no significant impact of ROR1-AS1 ectopic transfection on expression of cyclin D1 (*P* = 0.21) and ATM (*P* = 0.67), but the expression of the SOX11 was suppressed significantly 56% (*p* = 0.017) in Mino cells as compared to vector alone (Figure 5A). We then evaluated effect of ROR1-AS1 on tumor suppressor genes such as P14, P16 and P53 involved in the pathogenesis of MCL. QRT-PCR data indicated only modest effect on expression of p16 (*P* = 0.21), however, there was no effect observed on the expression of P14 and P53 (Figure 5B). These data suggest that ROR-AS1 might regulate expression of SOX11 and P16 in MCL cells.

### Effect of ROR-AS1 on drug sensitivity

**Figure 6:**
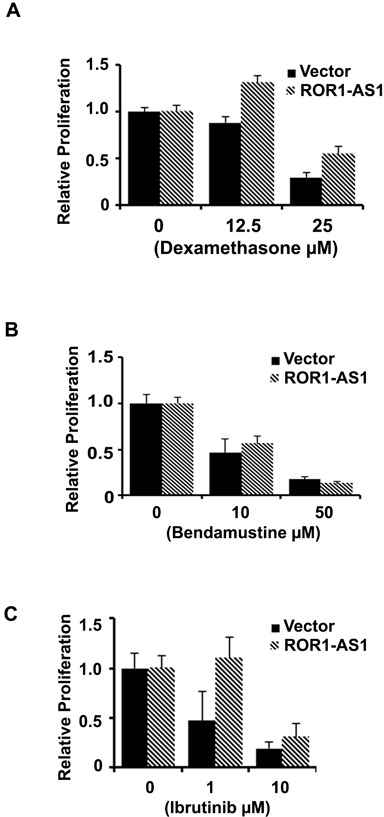
The effect of ROR1-AS1 overexpression on the sensitivity of MCL cells to chemotherapy drugs **A.-C.** Bar graphs showing effects of dexamethasone (A), bendamustine (B) and Ibrutinib (C) on proliferation (^3^H-thymidine incorporation) of Mino cells transfected with either empty vector or vector with ROR-AS1 expression cassette.

Some of the lncRNAs have been previously shown to render resistance to chemotherapy drugs [25]. We assessed the impact of lncRNA ROR-AS1 overexpression on sensitivity of MCL cells to the chemotherapeutic drugs bendamustine, dexamethasone and FDA approved, B cell receptor pathway drug ibrutinib. Results demonstrate that in Mino cells expressing empty vector treatment with 10 or 50 μM bendamustine decreased ^3^H-thymidine uptake by as 50 and 80%, respectively compared to vehicle control, ROR-AS1 overexpression had no significant impact on sensitivity to bendamustine (Figure 6A). Sensitivity to dexamethasone or ibrutinib in Mino cells led to a 50-75% decline in ^3^H-thymidine uptake in vector expressing cells. Interestingly proliferation inhibition by dexamethasone or ibrutinib was attenuated by overexpression of ROR-AS1 (Figure 6B-6C). This data suggested that lncRNA ROR-AS1 overexpression modulates sensitivity of MCL cells to therapeutic drugs and thus could influence the prognosis of MCL patients.

## DISCUSSION

Non-Hodgkin lymphoma is the seventh most common cancer in men and women. Mantle cell lymphoma (MCL) is a unique genetic and clinical subtype of NHL with a heterogeneous prognosis. Patients with MCL have a variable clinical course and in most cases disease eventually relapse, and median survival for MCL patients remains approximately 7 years [26, 27] Even though tremendous efforts have been made to optimize treatment, the progress in improvement in prognosis of MCL patients has been slow. New insights into the pathobiology of MCL, in addition to identify novel biomarkers and therapeutic targets are desperately needed. A better understanding of the mechanisms underlying the pathogenesis of MCL is important for improvement of therapy. Long noncoding RNAs (lncRNAs) such as HOTAIR and MALAT1 have been shown to be involved in the development of breast and lung cancer [28, 29], however, role of lncRNAs in lymphoma is still unknown. In this study, we analyzed lncRNA profile in MCL patients by next generation RNA sequencing and revealed for the first time that ROR1-AS1 lncRNA is highly upregulated in cells from MCL patients. Our analysis further showed that lncRNA ROR1-AS1 is predominantly localized in the nucleus, and physically associates with core proteins of PRC2 complex such as EZH2 and SUZ12. These results are consistent with prior reports suggesting role of HOTAIR lncRNA in chromatin reprograming by targeting PRC2 [29]. Our results further revealed that ROR1-AS1 overexpression is associated with increased cell proliferation suggesting a pivotal role for this lncRNA in chromatin reprogramming that contributes towards growth advantage in MCL cells. We demonstrate that EZH2 inhibition through pharmacological inhibitors abolished the interaction between ROR1-AS1 and EZH2, which further supports the notion that ROR1-AS1 interaction with EZH2 may impact activity of PRC2 complex. A number of lncRNAs has been shown to interact with EZH2 and regulate target gene expression. HOTAIR interacts with PRC2 and regulates HOXD locus and other target genes of EZH2 subunit [30]. Similarly, ANRIL lncRNA was found to recruit EZH2 to suppress INK4b/ARF/INK4a expression [28]. Thus, our results support the notion that LncRNA partner bound to EZH2 might help targeting of PRC2 complex to specific gene loci. Whole exome sequencing in MCL identified significantly mutated genes including known drivers of MCL malignancy such as ATM, Cyclin D1 and tumor suppressor gene TP53 [31]. However, overexpression of lncRNA ROR1-AS1 does not seem to impact expression of these transcripts. SOX11 is regarded as a prognostic marker in many different kinds of cancers especially the MCL [32–36]. Recently, transcriptional activity of SOX11 was implicated in biological processes including B-cell differentiation, tumor angiogenesis, and overall pathogenesis of MCL [37–40]. However, evidence in literature suggest that decreased expression of SOX11 associates with worse prognosis of MCL [35, 41]. While nuclear acculturation of SOX11 has been developed as a specific biomarker for MCL patients [8, 9, 42–44] but prognostic value of SOX11 and its role in disease progression is not well founded. Furthermore, the mechanism of transcriptional regulation of SOX11 in MCL remains unclear. Present study suggest that lncRNA ROR-AS1 in association with PRC2 complex influence transcription of SOX11.

In summary, our studies have identified for the first time the lncRNA profile in MCL cells and identified a ROR1-AS1 overexpression in MCL cells. The present work establishes that ROR1-AS1 mediates MCL growth through histone modification via EZH2. Such information provides new insights into the function and mechanisms of ROR1-AS1 in MCL pathogenesis. Whether lncRNA ROR1-AS1 is able to bind and regulate other histone modification complex such as chromatin modifier WHSC1 and MLL2 and induce gene suppression in an EZH2-independent mechanism remained to be investigated. Similarly, ROR1-AS1 may regulate multiple other targets, which may contribute to MCL pathogenesis or chemo-resistance remains the subject for future studies. Nevertheless, our results set a foundation for future discoveries of novel modulators of MCL pathogenesis, and likely to lead us to therapeutic targets and/or biomarkers of critical importance.

## MATERIALS AND METHODS

### Cell lines, drugs and MCL samples

The mantle cell lymphoma (MCL) cell lines Mino, Granta, JVM2 and Z138 were purchased from ATCC (Manassas, VA, USA). These cell lines were cultured in Roswell Park Memorial Institute medium (RPMI) supplemented with 10% fetal bovine serum (FBS). HEK-293T cell line purchased from Open Biosystem (Huntsville, AL, USA) was grown in the Dulbecco's Modified Eagle Medium supplemented with 10% FBS. Drugs dexamethasone and bendamustine were procured from Sigma-Aldrich, BTK inhibitor ibrutinib was purchased from Selleckchem. Mononuclear cells from MCL patients (*n* = 5) and normal control (lymph nodes) (*n* = 5) were provided by Mayo Clinic/Iowa Lymphoma SPORE.

### Antibodies

Antibodies to EZH2, EED, and SUZ12 were purchased from Abcam (Cambridge, MA USA).

### RNA-sequencing and bioinformatics analysis

RNA was extracted using Trizol reagent (Life Technologies) and sent to the Mayo Clinic Molecular Biology Core for RNA-sequencing to identify lncRNAs. The lncRNA expression was analyzed by bioinformatics tool ICQ-lincRNA developed at the Mayo Clinic. This computational pipeline integrates three consecutive steps: candidate transcript assembly, LincRNA identification, and annotation and visualization of LincRNAs.

### Plasmid construction

lncRNAs ROR1-AS1, AF127936.5 and RP11-12A2.3 were amplified using cDNA of Mino cells as template and cloned into vector IDT Smart (IDT). To construct the expression cassette, the respective lncRNA was extracted by restriction digestion of the carrier vector IDT Smart using BamHI (5′) and NotI (3′) and cloned in the expression vector pcDNA3.1 using T4 DNA Ligase (NEB, cat. no. M0202S). Plasmids were screened by restriction digestion, and subsequently confirmed by sequencing.

### Small interfering RNA (siRNA) and cell transfection

ROR1-AS1 siRNA was purchased from Dharmacon (Lafayette, CO, USA). Mino, Granta and JVM2 cells were transfected for 48 hours with ROR1-AS1 siRNA using Amaxa Nucleofector Kit (Lonza, Walkersville, MD, USA) as per instruction manual. For lncRNA overexpression Mino, Granta and JVM2 cells were transfected with 5 μg of plasmid (pcDNA3.1 empty vector or pcDNA3.1- ROR1-AS1, pcDNA3.1-AF127936.5, pcDNA3.1-RP11-12A2.3) using Amaxa Cell Line Nucleofector Kit and incubated for 24-48 hours prior to any analysis.

### Cell proliferation assay

Granta, Mino and JVM2 cells transfected with lncRNA ROR1-AS1 construct or empty vector were seeded into 96-well plate in 4 replicates (0.1 million/200 μl/well) and proliferation evaluated by ^3^H-thymidine incorporation assay as previously described [45].

### Extraction of cytoplasmic and nuclear RNA

Cytoplasmic and nuclear RNA were extracted using the Cytoplasmic and Nuclear RNA Purification Kit (Norgen, Thorold, Ontario, Canada) as per instruction manual. Briefly, 5-10 million cells were washed with PBS, centrifuged and cell pellet was subsequently lysed in 1 ml of lysis buffer and centrifuged at 14000 rpm at 4°C. Supernatant was used to extract cytoplasmic RNA, and the pellet processed for extraction of nuclear RNA.

### RT-PCR and quantitative RT-PCR (QRT-PCR)

RNA was extracted from 2 million cells with the RNeasy Mini Kit (QIAGEN, Germantown, MD, USA), cDNA synthesized with SuperScript III First-Strand Synthesis SuperMix (Invitrogen) and subjected to PCR or Quantitative PCR (Q-PCR) as previously described [18]. The primers used are:

ROR1-AS1-F 5′ CTGACGAAACACTGGAACTC 3′

ROR1-AS1-R 5′ GTCTGATTTGGTAGCTTGGATG 3′;

AC006196.1-F 5′ GATGACAGAGGATGTTCGAC 3′

AC006196.1-R 5′ CTGATGGAGGTATAGGAGTG 3′

Cyclin D1-F 5′ ACACTTCCTCTCCAAAATGCC 3′ and

Cyclin D1-R 5′ GAGGGCGGATTGGAAATGAAC 3′;

P16-m-F 5′ GAAGGTCCCTCAGACATCCCC 3′ and

P16-m-R 5′ CCCTGTAGGACCTTCGGTGAC 3′;

p14-m-F 5′ CCCTCGTGCTGATGCTACTG 3′ and

p14-m-R 5′ ACCTGGTCTTCTAGGAAGCGG 3′;

ATM-m-F 5′-CCAGGCAGGAATCATTCAG-3′ and

ATM-m-R 5′-CAATCCTTTTAAATAGACGGAAAGAA-3′;

P53-m-F 5′ CCCAAGCAATGGATGATTTGA 3′ and

P53-m-R 5′ GGCATTCTGGGAGCTTCATCT 3′;

SOX11-m-F 5′ GCGCTGTTTGAAGCTTGTCG 3′

SOX11-m-R 5′ TATCTCCACCAACTCCCTAG 3′.

### RNA-immunoprecipitation (RNA-IP)

RNP-IP was performed with Magna RIP RNA-Binding Protein Immuno-precipitation Kit (Millipore, cat #17-700) as previously described [18]*.* Briefly, 50 - 80 million cells were lysed in 200μl RIP lysis buffer before the lysate was immuno-precipitated with EZH2, EED, SUZ12 antibody (or IgG) attached to protein-A magnetic beads. After the digestion with proteinase K, the RNA was purified by phenol chloroform extraction. The RNA dissolved in 20 μl RNAse-free water for further analysis by RT-PCR.

### Statistical analyses

Unless stated otherwise data is presented as Mean ± SEM. Data was statistically analyzed using two-tailed Student's *t* test, *p* value < 0.05 considered significant.

## SUPPLEMENTARY MATERIALS FIGURES



## References

[1] Herrmann A, Hoster E, Zwingers T, Brittinger G, Engelhard M, Meusers P, Reiser M, Forstpointner R, Metzner B, Peter N, Wormann B, Trumper L, Pfreundschuh M (2009). Improvement of overall survival in advanced stage mantle cell lymphoma. Journal of Clinical Oncology.

[2] Geisler CH, Kolstad A, Laurell A, Jerkeman M, Raty R, Andersen NS, Pedersen LB, Eriksson M, Nordstrom M, Kimby E, Bentzen H, Kuittinen O, Lauritzsen GF (2012). Nordic MCL2 trial update: six-year follow-up after intensive immunochemotherapy for untreated mantle cell lymphoma followed by BEAM or BEAC + autologous stem-cell support: still very long survival but late relapses do occur. British Journal of Haematology.

[3] Jares P, Colomer D, Campo E (2012). Molecular pathogenesis of mantle cell lymphoma. The Journal of Clinical Investigation.

[4] Pinyol M, Hernandez L, Martinez A, Cobo F, Hernandez S, Bea S, Lopez-Guillermo A, Nayach I, Palacin A, Nadal A, Fernandez PL, Montserrat E, Cardesa A (2000). INK4a/ARF locus alterations in human non-Hodgkin's lymphomas mainly occur in tumors with wild-type p53 gene. The American Journal of Pathology.

[5] Pinyol M, Bea S, Pla L, Ribrag V, Bosq J, Rosenwald A, Campo E, Jares P (2007). Inactivation of RB1 in mantle-cell lymphoma detected by nonsense-mediated mRNA decay pathway inhibition and microarray analysis. Blood.

[6] Hernandez L, Fest T, Cazorla M, Teruya-Feldstein J, Bosch F, Peinado MA, Piris MA, Montserrat E, Cardesa A, Jaffe ES, Campo E, Raffeld M (1996). p53 gene mutations and protein overexpression are associated with aggressive variants of mantle cell lymphomas. Blood.

[7] Camacho E, Hernandez L, Hernandez S, Tort F, Bellosillo B, Bea S, Bosch F, Montserrat E, Cardesa A, Fernandez PL, Campo E (2002). ATM gene inactivation in mantle cell lymphoma mainly occurs by truncating mutations and missense mutations involving the phosphatidylinositol-3 kinase domain and is associated with increasing numbers of chromosomal imbalances. Blood.

[8] Ek S, Dictor M, Jerkeman M, Jirstrom K, Borrebaeck CA (2008). Nuclear expression of the non B-cell lineage Sox11 transcription factor identifies mantle cell lymphoma. Blood.

[9] Mozos A, Royo C, Hartmann E, De Jong D, Baro C, Valera A, Fu K, Weisenburger DD, Delabie J, Chuang SS, Jaffe ES, Ruiz-Marcellan C, Dave S (2009). SOX11 expression is highly specific for mantle cell lymphoma and identifies the cyclin D1-negative subtype. Haematologica.

[10] Nie L, Wu HJ, Hsu JM, Chang SS, Labaff AM, Li CW, Wang Y, Hsu JL, Hung MC (2012). Long non-coding RNAs: versatile master regulators of gene expression and crucial players in cancer. American journal of translational research.

[11] Holoch D, Moazed D (2015). RNA-mediated epigenetic regulation of gene expression. Nature Reviews Genetics.

[12] Mercer TR, Dinger ME, Mattick JS (2009). Long non-coding RNAs: insights into functions. Nature Reviews Genetics.

[13] Tripathi V, Ellis JD, Shen Z, Song DY, Pan Q, Watt AT, Freier SM, Bennett CF, Sharma A, Bubulya PA, Blencowe BJ, Prasanth SG, Prasanth KV (2010). The nuclear-retained noncoding RNA MALAT1 regulates alternative splicing by modulating SR splicing factor phosphorylation. Molecular Cell.

[14] Gong C, Maquat LE (2011). lncRNAs transactivate STAU1-mediated mRNA decay by duplexing with 3′ UTRs via Alu elements. Nature.

[15] Gumireddy K, Li A, Yan J, Setoyama T, Johannes GJ, Orom UA, Tchou J, Liu Q, Zhang L, Speicher DW, Calin GA, Huang Q (2013). Identification of a long non-coding RNA-associated RNP complex regulating metastasis at the translational step. The EMBO Journal.

[16] Carrieri C, Cimatti L, Biagioli M, Beugnet A, Zucchelli S, Fedele S, Pesce E, Ferrer I, Collavin L, Santoro C, Forrest AR, Carninci P, Biffo S (2012). Long non-coding antisense RNA controls Uchl1 translation through an embedded SINEB2 repeat. Nature.

[17] Yoon JH, Abdelmohsen K, Srikantan S, Yang X, Martindale JL, De S, Huarte M, Zhan M, Becker KG, Gorospe M (2012). LincRNA-p21 suppresses target mRNA translation. Molecular Cell.

[18] Hu G, Lou Z, Gupta M (2014). The long non-coding RNA GAS5 cooperates with the eukaryotic translation initiation factor 4E to regulate c-Myc translation. PLoS One.

[19] Fatica A, Bozzoni I (2014). Long non-coding RNAs: new players in cell differentiation and development. Nature Reviews Genetics.

[20] Esteller M (2011). Non-coding RNAs in human disease. Nature Reviews Genetics.

[21] Niemann CU, Wiestner A (2013). B-cell receptor signaling as a driver of lymphoma development and evolution. Seminars in Cancer Biology.

[22] Khalil AM, Guttman M, Huarte M, Garber M, Raj A, Rivea Morales D, Thomas K, Presser A, Bernstein BE, van Oudenaarden A, Regev A, Lander ES, Rinn JL (2009). Many human large intergenic noncoding RNAs associate with chromatin-modifying complexes and affect gene expression. Proceedings of the National Academy of Sciences of the United States of America.

[23] Verma SK, Tian X, LaFrance LV, Duquenne C, Suarez DP, Newlander KA, Romeril SP, Burgess JL, Grant SW, Brackley JA, Graves AP, Scherzer DA, Shu A (2012). Identification of Potent, Selective, Cell-Active Inhibitors of the Histone Lysine Methyltransferase EZH2. ACS Med Chem Lett.

[24] Jares P, Colomer D, Campo E (2007). Genetic and molecular pathogenesis of mantle cell lymphoma: perspectives for new targeted therapeutics. Nature Reviews Cancer.

[25] Malek E, Jagannathan S, Driscoll JJ (2014). Correlation of long non-coding RNA expression with metastasis, drug resistance and clinical outcome in cancer. Oncotarget.

[26] Hoster E, Klapper W, Hermine O, Kluin-Nelemans HC, Walewski J, van Hoof A, Trneny M, Geisler CH, Di Raimondo F, Szymczyk M, Stilgenbauer S, Thieblemont C, Hallek M (2014). Confirmation of the mantle-cell lymphoma International Prognostic Index in randomized trials of the European Mantle-Cell Lymphoma Network. J Clin Oncol.

[27] Rummel MJ, Niederle N, Maschmeyer G, Banat GA, von Grunhagen U, Losem C, Kofahl-Krause D, Heil G, Welslau M, Balser C, Kaiser U, Weidmann E, Durk H (2013). Bendamustine plus rituximab versus CHOP plus rituximab as first-line treatment for patients with indolent and mantle-cell lymphomas: an open-label, multicentre, randomised, phase 3 non-inferiority trial. Lancet.

[28] Yap KL, Li S, Munoz-Cabello AM, Raguz S, Zeng L, Mujtaba S, Gil J, Walsh MJ, Zhou MM (2010). Molecular interplay of the noncoding RNA ANRIL and methylated histone H3 lysine 27 by polycomb CBX7 in transcriptional silencing of INK4a. Mol Cell.

[29] Gupta RA, Shah N, Wang KC, Kim J, Horlings HM, Wong DJ, Tsai MC, Hung T, Argani P, Rinn JL, Wang Y, Brzoska P, Kong B (2010). Long non-coding RNA HOTAIR reprograms chromatin state to promote cancer metastasis. Nature.

[30] Rinn JL, Kertesz M, Wang JK, Squazzo SL, Xu X, Brugmann SA, Goodnough LH, Helms JA, Farnham PJ, Segal E, Chang HY (2007). Functional demarcation of active and silent chromatin domains in human HOX loci by noncoding RNAs. Cell.

[31] Bea S, Valdes-Mas R, Navarro A, Salaverria I, Martin-Garcia D, Jares P, Gine E, Pinyol M, Royo C, Nadeu F, Conde L, Juan M, Clot G (2013). Landscape of somatic mutations and clonal evolution in mantle cell lymphoma. Proc Natl Acad Sci U S A.

[32] Walter RF, Mairinger FD, Werner R, Ting S, Vollbrecht C, Theegarten D, Christoph DC, Zarogoulidis K, Kurt Werner S, Zarogoulidis P, Wohlschlaeger J (2015). SOX4, SOX11 and PAX6 mRNA expression was identified as a (prognostic) marker for the aggressiveness of neuroendocrine tumors of the lung by using next-generation expression analysis (NanoString). Future Oncology.

[33] Roisman A, Stanganelli C, Nagore VP, Richardson GV, Scassa ME, Bezares RF, Cabrejo M, Slavutsky I (2015). SOX11 expression in chronic lymphocytic leukemia correlates with adverse prognostic markers. Tumour Biology.

[34] Nordstrom L, Sernbo S, Eden P, Gronbaek K, Kolstad A, Raty R, Karjalainen ML, Geisler C, Ralfkiaer E, Sundstrom C, Laurell A, Delabie J, Ehinger M (2014). SOX11 and TP53 add prognostic information to MIPI in a homogenously treated cohort of mantle cell lymphoma—a Nordic Lymphoma Group study. British Journal of Haematology.

[35] Nygren L, Baumgartner Wennerholm S, Klimkowska M, Christensson B, Kimby E, Sander B (2012). Prognostic role of SOX11 in a population-based cohort of mantle cell lymphoma. Blood.

[36] Meggendorfer M, Kern W, Haferlach C, Haferlach T, Schnittger S (2013). SOX11 overexpression is a specific marker for mantle cell lymphoma and correlates with t(11;14) translocation, CCND1 expression and an adverse prognosis. Leukemia.

[37] Vegliante MC, Palomero J, Perez-Galan P, Roue G, Castellano G, Navarro A, Clot G, Moros A, Suarez-Cisneros H, Bea S, Hernandez L, Enjuanes A, Jares P (2013). SOX11 regulates PAX5 expression and blocks terminal B-cell differentiation in aggressive mantle cell lymphoma. Blood.

[38] Palomero J, Vegliante MC, Rodriguez ML, Eguileor A, Castellano G, Planas-Rigol E, Jares P, Ribera-Cortada I, Cid MC, Campo E, Amador V (2014). SOX11 promotes tumor angiogenesis through transcriptional regulation of PDGFA in mantle cell lymphoma. Blood.

[39] Kuo PY, Leshchenko VV, Fazzari MJ, Perumal D, Gellen T, He T, Iqbal J, Baumgartner-Wennerholm S, Nygren L, Zhang F, Zhang W, Suh KS, Goy A (2015). High-resolution chromatin immunoprecipitation (ChIP) sequencing reveals novel binding targets and prognostic role for SOX11 in mantle cell lymphoma. Oncogene.

[40] Wang X, Bjorklund S, Wasik AM, Grandien A, Andersson P, Kimby E, Dahlman-Wright K, Zhao C, Christensson B, Sander B (2010). Gene expression profiling and chromatin immunoprecipitation identify DBN1, SETMAR and HIG2 as direct targets of SOX11 in mantle cell lymphoma. PLoS One.

[41] Lu TX, Li JY, Xu W (2013). The role of SOX11 in mantle cell lymphoma. Leuk Res.

[42] Zeng W, Fu K, Quintanilla-Fend L, Lim M, Ondrejka S, Hsi ED (2012). Cyclin D1-negative blastoid mantle cell lymphoma identified by SOX11 expression. The American Journal of Surgical Pathology.

[43] Wang X, Asplund AC, Porwit A, Flygare J, Smith CI, Christensson B, Sander B (2008). The subcellular Sox11 distribution pattern identifies subsets of mantle cell lymphoma: correlation to overall survival. British Journal of Haematology.

[44] Dictor M, Ek S, Sundberg M, Warenholt J, Gyorgy C, Sernbo S, Gustavsson E, Abu-Alsoud W, Wadstrom T, Borrebaeck C (2009). Strong lymphoid nuclear expression of SOX11 transcription factor defines lymphoblastic neoplasms, mantle cell lymphoma and Burkitt's lymphoma. Haematologica.

[45] Gupta M, Ansell SM, Novak AJ, Kumar S, Kaufmann SH, Witzig TE (2009). Inhibition of histone deacetylase overcomes rapamycin-mediated resistance in diffuse large B-cell lymphoma by inhibiting Akt signaling through mTORC2. Blood.

