# Supplementary material for: Long non-coding RNA profile in mantle cell lymphoma identifies a functional lncRNA ROR1-AS1 associated with EZH2/PRC2 complex
Source: Oncotarget. 2017 May 17;8(46):80223–34. doi: 10.18632/oncotarget.17956 (PMC5655192; doi:10.18632/oncotarget.17956)
Supplement: Supplementary file 1 [file oncotarget-08-80223-s001.pdf]

Long non-coding RNA profile in mantle cell lymphoma identifies a functional lncRNA ROR1-AS1 associated with EZH2/PRC2 complex

SUPPLEMENTARY MATERIALS

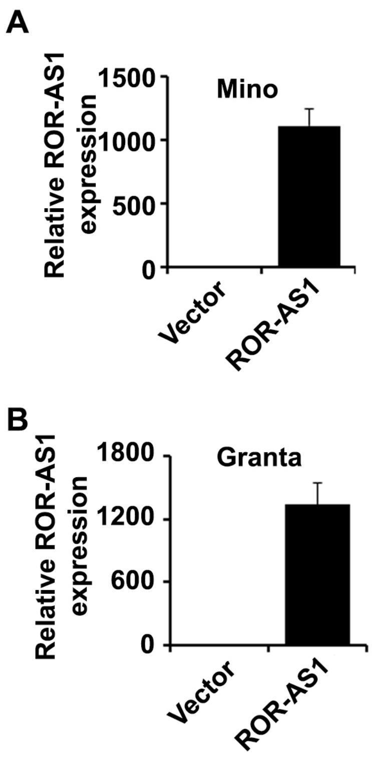

Supplementary Figure 1: (A.-B.) Overexpression of lncRNAs ROR-AS1 in Mino and Granta cells by transient transfection.

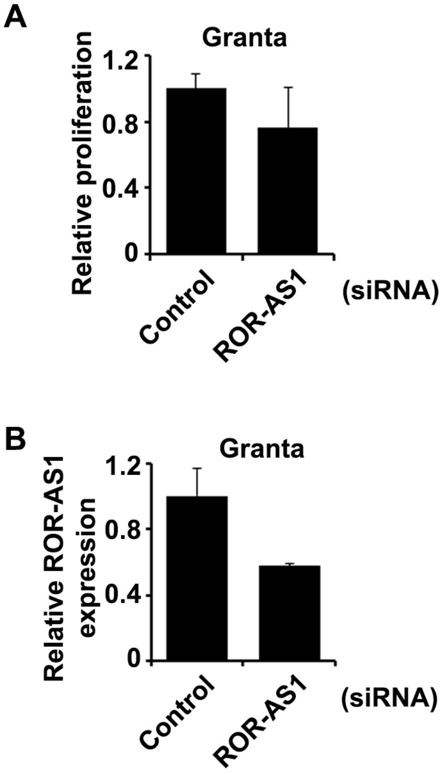

Supplementary Figure 2: (**A**) Effect of knockdown of ROR-AS1 on proliferation of Granta cells, (**B**) knockdown of ROR-AS1 in Granta cells
